# Efficient Killing of Murine Pluripotent Stem Cells by Natural Killer (NK) Cells Requires Activation by Cytokines and Partly Depends on the Activating NK Receptor NKG2D

**DOI:** 10.3389/fimmu.2017.00870

**Published:** 2017-07-26

**Authors:** Carina Gröschel, Daniela Hübscher, Jessica Nolte, Sebastian Monecke, André Sasse, Leslie Elsner, Walter Paulus, Claudia Trenkwalder, Bojan Polić, Ahmed Mansouri, Kaomei Guan, Ralf Dressel

**Affiliations:** ^1^Institute of Cellular and Molecular Immunology, University Medical Center Göttingen, Göttingen, Germany; ^2^DZHK (German Center for Cardiovascular Research), Göttingen, Germany; ^3^Department of Cardiology and Pneumology, University Medical Center Göttingen, Göttingen, Germany; ^4^Institute of Human Genetics, University Medical Center Göttingen, Göttingen, Germany; ^5^Department of Clinical Neurophysiology, University Medical Center Göttingen, Göttingen, Germany; ^6^Department of Neurosurgery, University of Göttingen, Göttingen, Germany; ^7^Department of Histology and Embryology, Faculty of Medicine, University of Rijeka, Rijeka, Croatia; ^8^Department of Molecular Cell Biology, Max Planck Institute for Biophysical Chemistry, Göttingen, Germany; ^9^Institute of Pharmacology and Toxicology, Technische Universität Dresden, Dresden, Germany

**Keywords:** teratoma, autologous transplantation, embryonic stem cells, induced pluripotent stem cells, multipotent adult germline stem cells, natural killer cells, cytokine-activated natural killer cells, natural killer receptor ligands

## Abstract

Natural killer (NK) cells play an important role as cytotoxic effector cells, which scan the organism for infected or tumorigenic cells. Conflicting data have been published whether NK cells can also kill allogeneic or even autologous pluripotent stem cells (PSCs) and which receptors are involved. A clarification of this question is relevant since an activity of NK cells against PSCs could reduce the risk of teratoma growth after transplantation of PSC-derived grafts. Therefore, the hypothesis has been tested that the activity of NK cells against PSCs depends on cytokine activation and specifically on the activating NK receptor NKG2D. It is shown that a subcutaneous injection of autologous iPSCs failed to activate NK cells against these iPSCs and can give rise to teratomas. In agreement with this result, several PSC lines, including two iPSC, two embryonic stem cell (ESC), and two so-called multipotent adult germline stem cell (maGSC) lines, were largely resistant against resting NK cells although differences in killing were found at low level. All PSC lines were killed by interleukin (IL)-2-activated NK cells, and maGSCs were better killed than the other PSC types. The PSCs expressed ligands of the activating NK receptor NKG2D and NKG2D-deficient NK cells from *Klrk1^−^*^/^*^−^* mice were impaired in their cytotoxic activity against PSCs. The low-cytotoxic activity of resting NK cells was almost completely dependent on NKG2D. The cytotoxic activity of IL-2-activated NKG2D-deficient NK cells against PSCs was reduced, indicating that also other activating receptors on cytokine-activated NK cells must be engaged by ligands on PSCs. Thus, NKG2D is an important activating receptor involved in killing of murine PSCs. However, NK cells need to be activated by cytokines before they efficiently target PSCs and then also other NK receptors become relevant. These features of NK cells might be relevant for transplantation of PSC-derived grafts since NK cells have the capability to kill undifferentiated cells, which might be present in grafts in trace amounts.

## Introduction

Pluripotent stem cells (PSCs) hold a great potential for the therapy of diseases, which are currently incurable, including heart failure, Parkinson’s disease, and macular degeneration (1), since cellular grafts or even complex tissues can be generated *in vitro* by directed differentiation of these cells (2). Clinical trials to evaluate the therapeutic potential of PSCs have been initiated (3, 4), and first encouraging results of studies using human embryonic stem cells (ESCs) (5) as source of grafts to treat macular degeneration (6–8) and heart failure (9) have been published.

Embryonic stem cell-derived grafts have to be transplanted in an allogeneic setting and, therefore, the rejection of ESC-derived allografts is a challenge for these new therapies. Although immunosuppressive therapy can efficiently prevent the rejection of allogenic organs, the persistent use of immunosuppressive drugs is associated with problems of toxicity and increased risks of infections and cancer. Therefore, other PSC types, which can be obtained from adult cells including so-called multipotent adult germline stem cells (maGSCs) (10) and induced pluripotent stem cells (iPSCs) (11–14) gained attention as potential source of autologous grafts. Autologous grafts should be transplantable without the need of immunosuppression, and recently, first results on the transplantation of retinal epithelial cells derived from autologous iPSCs have been reported (15). However, it is still debated whether grafts derived from iPSCs can be immunogenic in syngeneic or autologous recipients (16, 17). While terminally differentiated grafts appear to be tolerated as expected (18–20), it has been reported that therapeutically relevant *in vitro*-differentiated human iPSC-derived grafts were at risk to be rejected in mice “humanized” with an “autologous immune system” depending on the direction of differentiation (21).

A further concern associated with the transplantation of PSC-derived grafts is the risk of tumorigenicity (22, 23). Notably, the risk of tumorigenicity might be even higher for iPSCs than ESCs due to mutations preexisting in the reprogrammed somatic cells, introduced during the reprogramming process or resulting from an increased genomic instability of iPSCs (24–26). However, all types of PSCs can give rise to teratomas. In immunodeficient mice, as few as 2 murine or 245 human ESCs were reported to elicit teratoma growth (27, 28). ESCs can also form teratomas in immunocompetent syngeneic recipients (29), although higher cell numbers appear to be required than in immunodeficient recipients (22, 30, 31). The risk of teratoma growth upon transplantation of autologous iPSCs into immunocompetent recipients has not been assessed to our knowledge. However, in allogeneic immunocompetent hosts, PSCs are usually rejected and do not form teratomas (22, 31) although exceptions from this rule have been reported (32).

Natural killer (NK) cells have important effector functions such as cytotoxicity and cytokine production. The activity of NK cells is regulated by a plethora of germ-line encoded receptors that recognize ligands on target cells (33). NK receptors include activating and inhibitory members. Inhibitory receptors of the Ly49 family in mice or the killer cell immunoglobulin-like receptor (KIR) family in humans interact with classical major histocompatibility complex (MHC) class Ia molecules, which are constitutively expressed on most cells (34, 35). In contrast, many activating receptors, including NKG2D (36), recognize ligands, which are mostly absent from healthy cells but become induced upon stress such as virus infection or malignant transformation (37). Classically, NK cells have been described as cells that, unlike cytotoxic T cells, do not require activation to exert cytotoxicity. However, stimulation with interleukin 2 (IL-2), IL-12, IL-15, IL-18, or combinations of these cytokines can markedly increase the activity of NK cells (38). While some targets such as the classical human NK cell target cell line K562 are efficiently killed also by resting NK cells, killing of others requires cytokine-activated NK cells (39).

Conflicting results have been published regarding the susceptibility of ESCs toward NK cells. Some studies have reported resistance or very low susceptibility (40, 41) while others found ESCs to be vulnerable to NK cells (42, 43), and this might be due to differences in the activation status of the NK cells in these studies. We have shown previously that murine PSCs, including ESCs, iPSCs, and maGSCs, are targets for IL-2-activated NK cells *in vitro* (31, 44) and that NK cells can impair or even suppress the teratoma growth upon transplantation of the PSCs *in vivo* (22, 44, 45). Murine PSCs are targets for allogeneic and syngeneic NK cells because they do not express MHC class I antigens, which serve as ligands for inhibitory NK receptors, at least at a level detectable by flow cytometry (31, 44, 46), but they do express ligands for activating NK receptors, such as NKG2D and DNAM-1 (31, 42, 44). Similar expression patterns of ligands for activating NK receptors as on murine PSCs were found on human iPSCs, which were also targets of IL-2-activated allogeneic and autologous NK cells (39). Notably, human ESCs and iPSCs, in contrast to the respective murine cells, do express MHC class I molecules although in lower amounts than most differentiated cell types (39, 47). We have previously shown by inhibition experiments that killing of murine PSCs by IL-2-activated NK cells was partly dependent on NKG2D while killing of human iPSCs was more dependent on DNAM-1 (31, 39, 44).

Differentiation of PSCs usually increases the expression of MHC class I molecules (48, 49) and decreases the expression of NKG2D ligands (31, 44) and consequently *in vitro*-differentiated cells acquire resistance against NK cells (44). Thus, NK cells have the capability to target undifferentiated PSCs while ignoring differentiated cells. NK cells might, therefore, increase the safety of transplantations of PSC-derived grafts because they potentially increase the number of undifferentiated cells that can be tolerated in a graft. However, it needs to be clarified whether NK cells need activation by cytokines to target PSCs and which receptor–ligand interactions between NK cells and PSCs are important.

In this study, we set out to demonstrate that murine iPSCs can indeed form teratomas upon truly autologous transplantation and we tested whether NK cells become activated *in vivo* upon transplantation. Moreover, to resolve previous conflicting results, we directly compared the cytotoxic activity of resting and IL-2-activated NK cells against PSCs, including ESCs, iPSCs, and maGSCs and analyzed the role of the activating NK receptor NKG2D by comparing wild-type and NKG2D-deficient NK cells.

## Materials and Methods

### Mouse Strains

C57BL/6, 129Sv, and NKG2D-deficient *Klrk1^−^*^/^*^−^* mice (50) as well as immunodeficient *Rag2^−^*^/^*^−^* and SCID/beige mice (C.B-17/IcrHsd-scid-bg) were bred in the central facility for animal experimentation at the University Medical Center Göttingen under specific pathogen-free conditions in individually ventilated cages and in a 12 h light–dark cycle. The *Klrk1^−^*^/^*^−^* and *Rag2^−^*^/^*^−^* mice were on a C57BL/6 background. All animal experiments had been approved by the local government (Niedersächsisches Landesamt für Verbraucherschutz und Lebensmittelsicherheit) and were carried out in compliance with German and EU legislation (Directive 2010/63/EU).

### Stem Cell Lines and Cell Culture

#### Generation of New iPSC Lines

Tail biopsies were taken aseptically, cut into pieces, and incubated in dispase solution (2 mg/ml, Thermo Fisher Scientific, Waltham, MA, USA) for 20 min at 37°C, washed with phosphate-buffered saline (PBS), and incubated again for 20 min at 37°C in 0.05% trypsin with 0.005% EDTA. After being washed, the isolated cells were cultured in Dulbecco’s modified Eagle’s medium (DMEM)/F12 plus GlutaMAX™ (Thermo Fisher Scientific) supplemented with 10% fetal calf serum (FCS; selected batch, Lonza, Basel, Switzerland), 1× N2 supplement (Thermo Fisher Scientific), 1× non-essential amino acids (NEAA; Thermo Fisher Scientific), 50 µM β-mercaptoethanol (β-ME; Promega, Mannheim, Germany), 0.3 µg/ml hydrocortisone (Sigma-Aldrich, Munich, Germany), 10 ng/ml human epidermal growth factor (Peprotech, Rocky Hill, NJ, USA), 10 ng/ml human basic fibroblast growth factor (hbFGF, Peprotech), and 100 U/ml penicillin plus 100 µg/ml streptomycin (Thermo Fisher Scientific). Outgrowing fibroblasts were transduced with the STEMCCA virus (pHAGE2-EF1αFull-hOct4-F2A-hKlf4-IHRES-hSox2-P2A-hc-Myc-W-loxP), which was kindly provided by Dr. Darrell Kotton, University of Boston. Cells transduced by the STEMCCA virus express the human pluripotency genes *OCT4, KLF4, SOX2*, and *cMYC*. Fibroblasts were transduced at a multiplicity of infection of 1 in 24-well-plates with 700 µl FIII medium [minimum essential medium α (MEMα; Thermo Fisher Scientific) with 4.5% FCS, 4.5% knockout serum replacement (Thermo Fisher Scientific), 50 µM β-ME, 0.35% d-(+)-glucose (Sigma-Aldrich), and 1,000 U/ml leukemia inhibitory factor (LIF; Merck Millipore, Darmstadt, Germany)] and 5 µg/ml polybrene (hexadimethrine bromide, Sigma-Aldrich). After culture for 24 h at 37°C, the virus was washed away and the cells were cultured for 5–7 days. Then, the cells were seeded on mitomycin C-inactivated mouse embryonic feeder cells in DMEM (Thermo Fisher Scientific) supplemented with 15% FCS, 2 mM glutamine (Thermo Fisher Scientific), 1× NEAA, 50 µM β-ME, and 1,000 U/ml LIF as described previously (10). Single colonies of reprogrammed cells were picked to obtain stable cell lines for further analysis.

#### Culture of Stem Cell Lines

The ESC line MPI-II derived from a 129Sv mouse at the Max Planck Institute for Biophysical Chemistry (Göttingen, Germany) has been extensively characterized previously including the demonstration of pluripotency by teratoma assays (31). After transfer of breeders of that colony to the University Medical Center Göttingen, a new ESC line (ESC BTL1) has been generated from a 129Sv embryo as described (51). In parallel, two iPSC lines were generated as described above, one from the same 129Sv colony (iPSC129Sv) and one from a C57BL/6J mouse (Janvier Labs, Le Genest Saint Isle, France). The maGSC lines, maGSC 129Sv, and maGSC C57BL/6 have been described earlier (10) and their pluripotency has also been demonstrated in teratoma assays (44). The well-characterized ESC R1 cell line (52) has been used as positive control for expression of pluripotency genes in quantitative PCR (qPCR) experiments.

Before the PSC lines were used as target cells for NK cells, they were cultured on Geltrex-coated cell culture plates (333 µg/T25 flask), prepared according to manufacturer’s instructions (Thermo Fisher Scientific) in DMEM supplemented with 10% FCS, 1× NEAA, 50 µM β-ME, and 1,000 U/ml LIF. For harvesting at 80% confluence, the cells were incubated in stem cell trypsin (0.25% trypsin, 0.02% EDTA, 0.1% glucose, 0.3% Tris–HCl in PBS) for several minutes at 37°C until dissociation of stem cell colonies occurred. A single cell suspension was prepared by diluting cells in 10 volumes of stem cell medium. Stem cells were tested by PCR assays prior to experiments to exclude fungal, bacterial, and mycoplasmal infections.

#### Culture of YAC-1 Cells

The murine T-lymphoma cell line YAC-1 (H2^a^), which was used as positive control for the cytotoxic activity of NK cells, was maintained in DMEM supplemented with 10% FCS, 2 mM glutamine, 1 mM sodium pyruvate, 50 µM β-ME, 100 U/ml penicillin, and 100 µg/ml streptomycin.

### Pluripotency Assays

#### Alkaline Phosphatase Staining and Immunofluorescence Staining of Pluripotency Marker Proteins

The alkaline phosphatase activity of PSCs was detected using a kit according to the manufacturer’s instructions (Sigma-Aldrich). Expression of marker proteins of PSCs (LIN28, NANOG, and SSEA1) was analyzed by immunofluorescence staining. The stem cells were grown on cover-slips coated by 0.1% gelatin. The cells were fixed with 4% paraformaldehyde in PBS before blocking in 1% bovine serum albumin solution. For detection of NANOG, the cells were permeabilized (0.1% Triton X-100, Sigma-Aldrich) for 10 min before incubation with the primary antibody (Ab). The primary Abs (Table S1 in Supplementary Material) were added in 100 µl PBS to the cover-slips and incubated for 1 h at 37°C in a humidified chamber. Afterward, the cover-slips were washed three times with PBS before the secondary Ab was added. After incubation for 1 h at 37°C, the cover-slips were washed again before nuclei were stained with 4′,6-diamidino-2-phenylindol (DAPI, 0.4 µg/ml, Sigma-Aldrich) for 10 min. The samples were mounted in Vectashield mounting medium (Vector Laboratories, Cambridgeshire, UK) and analyzed with the fluorescence microscope Axio Observer Z1 (Zeiss, Jena, Germany) and Axio Vision 4.6 software.

#### Immunoblotting for Detection of Pluripotency Marker Proteins

In ESC BTL1 cells, the pluripotency marker proteins OCT4, SALL4, SOX2, KLF4, and ZPF206 were detected by immunoblotting and α-tubulin served as loading control. The antibodies used are given in Table S1 in Supplementary Material, and the immunoblotting was done as described previously (53).

#### *In Vitro* Differentiation of iPSCs

For *in vitro* differentiation of iPSCs, the hanging drop method was applied. Hanging drops containing 300 cells in 20 µl Iscove’s modified Dulbecco’s medium plus GlutaMAX™ (Thermo Fisher Scientific) supplemented with 20% FCS, 1× NEAA, 450 µM α-Monothioglycerol (Sigma-Aldrich) were cultured for 2 days. In this time, embryoid bodies had formed and were subsequently transferred into Petri dishes for a 3-day suspension culture period before transfer to gelatinized (0.1%) cell culture dishes for further 5, 15, or 25 days. Medium was exchanged every second or third day. The cells were harvested at the indicated time points, and expression of genes that indicate differentiation into the three germ layers was analyzed.

#### Reverse Transcription Polymerase Chain Reaction (RT-PCR) and qPCR for Expression Analysis of Pluripotency and Differentiation Genes

Total RNA was extracted from PSCs or *in vitro*-differentiated cells, treated with DNase I to avoid contamination with genomic DNA, and used for cDNA synthesis as described previously (46). The analyzed genes and the primer pairs used are given in Table S2 in Supplementary Material. The iPSC lines and their differentiation products were analyzed by qualitative RT-PCR as described previously (10, 54). The expression of pluripotency genes in ESC BTL1 cells was determined by qPCR as described before (53).

#### Teratoma Assays

The PSCs were injected in 100 µl PBS subcutaneously into the flank of the mice. Tumor growth was monitored by palpation and size was recorded using linear calipers. Animals were sacrificed after 3 months or when a tumor volume of 1 cm^3^ was reached. The tumor volume was calculated by the formula V = πabc/2, where *a, b*, and *c* are the orthogonal diameters. Autopsies of all animals were performed and tumor tissues were immediately frozen in liquid nitrogen or directly placed in phosphate-buffered formalin (30 mM NaH_2_PO_4_, 40 mM Na_2_HPO_4_, 4% formalin) for 16 h before being embedded in paraffin. Tissue sections (2.5 µm) were stained with hematoxylin and eosin for histological examination.

### NK Cells and Cytotoxicity Assay

Natural killer cells were isolated from spleens of C57BL/6 wild-type and NKG2D-deficient *Klrk1^−^*^/^*^−^* mice by magnetic-activated cell sorting using a kit for negative selection (mouse NK Cell Isolation Kit II; Miltenyi Biotec; Bergisch-Gladbach, Germany) according to the manufacturer’s protocol. The proportion of NK cells in the spleens and the purity of the isolated CD49b^+^CD3^−^ NK cells were always determined by flow cytometry and were similar in wild-type and NKG2D-deficient mice (Figure S1 in Supplementary Material). In parallel, we always confirmed the expression of NKG2D on CD49b^+^ wild-type NK cells and the NKG2D-deficiency on *Klrk1^−^*^/^*^−^* CD49b^+^ NK cells (data not shown). The purified NK cells were either directly used as cytotoxic effector cells (day 0) or after stimulation for 4 days with 10 ng/ml mouse IL-2 (Immunotools, Friesoythe, Germany). The cytotoxic activity of the NK cells against the PSCs and YAC-1 control cells was measured in ^51^Cr-release assays as described previously (45). The ^51^Cr-labeled target cells were exposed to the NK cells in triplicates at several effector to target (E:T) ratios for 4 h. The E:T ratios always indicate the ratio of CD49b^+^CD3*^−^* effector cells to target cells. Spontaneous release of ^51^Cr was determined by incubation of target cells in the absence of effector cells and specific lysis was calculated by subtracting the spontaneous ^51^Cr-release.

### Flow Cytometry

Flow cytometry was performed on a FACS Calibur flow cytometer (BD Biosciences, Heidelberg, Germany) using CellQuestPro data acquisition and analysis software. The Abs used for flow cytometry are described in Table S3 in Supplementary Material. Isotype controls were used for directly labeled monoclonal antibodies (mAbs). For staining, 5 × 10^5^ cells were incubated in 100 µl PBS with 1 µg of the respective primary mAb for 30 min at 4°C before washing with PBS. To detect the unlabeled mAbs, the cells were incubated subsequently in 100 µl PBS with 1 µl of a fluorescein isothiocyanate-labeled goat anti-rat IgG Ab (for anti-RAE-1, anti-MULT-1, and anti-H60). In these experiments, cells stained with the secondary reagent only served as control. The percentage of positive cells expressing the analyzed molecule and their mean fluorescence intensity (MFI) was determined by subtracting the values of the appropriate controls.

### Statistics

Results are shown as mean with SD or as mean with SEM for cytotoxicity assays. The data were evaluated with the SPSS software (IBM, Armonk, NY, USA). Analyses of variance (ANOVA) adjusted for effector to target (E:T) ratios were used to evaluate the cytotoxicity data and Bonferroni tests were selected as *post hoc* tests. A *P*-value of ≤ 0.05 in two-sided tests was considered significant.

## Results

### No NK Cell Activation against Autologous iPSCs *In Vivo*

**Figure 1:**
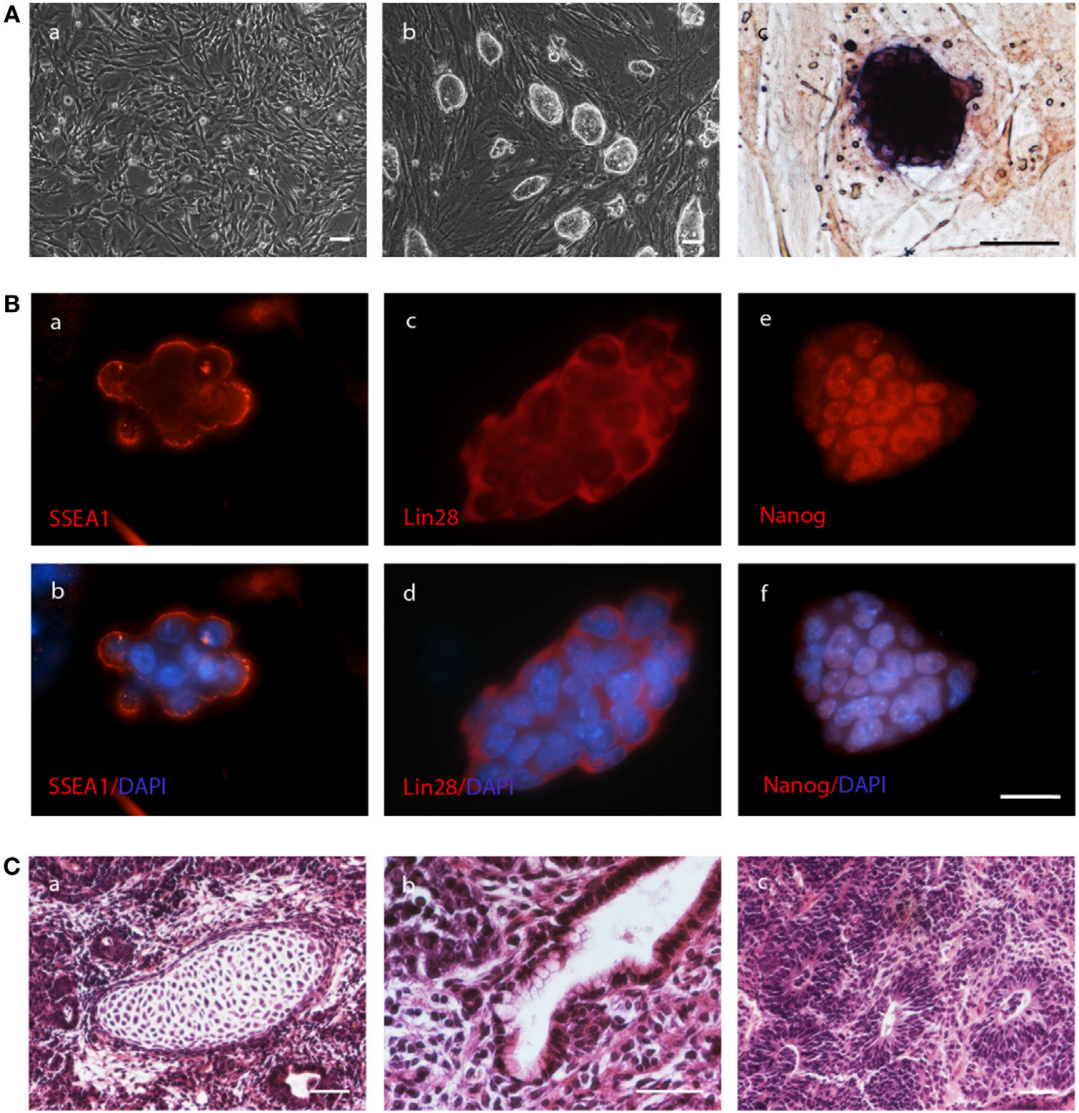
Autologous induced pluripotent stem cell (iPSC) lines are pluripotent and can give rise to teratomas. **(A)** Fibroblasts (a) isolated from tail tip biopsies of C57BL/6J mice gave rise to colonies of iPSCs (b) upon transduction with the reprogramming vector. The colonies were positive for alkaline phosphatase (c). The scale bars indicate 50 µm. **(B)** Colonies of the iPSCs also expressed the pluripotency marker proteins SSEA1 (a, b), LIN28 (c, d), and NANOG (e, f) as demonstrated by immunofluorescence staining alone (a, c, e) or in combination with DAPI to counterstain nuclei (b, d, f). The scale bar indicates 25 µm. **(C)** Cells of the iPSC line 1–2 were subcutaneously injected into the mouse that donated the fibroblast and a teratoma was obtained after 35 days. The mesodermal differentiation into cartilage (a), endodermal differentiation into intestinal epithelium (b), and ectodermal differentiation into neural rosettes (c) is shown. The scale bars indicate 100 µm.

We generated fibroblasts from the tail tips of five C57BL/6J mice at the age of 11 days. They were transduced with the STEMCCA virus for reprogramming into iPSCs. Colonies of cells with PSC morphology, which were positive for alkaline phosphatase, were obtained from all transductions as exemplified in Figure 1A. Single colonies were picked and iPSC lines were established from the five mice. These lines expressed the endogenous pluripotency genes *Oct4, Sox2, Nanog*, and *Lin28* (Figure S2A in Supplementary Material). They were positive for the pluripotency markers SSEA1, LIN28, and NANOG at the protein level as shown by immunofluorescence staining (Figure 1B). After *in vitro* differentiation via the hanging drop method, marker genes for differentiated cells were expressed, including *Afp* for the endodermal lineage, *Mash1* for the ectodermal linage, and *Flk1* for the mesodermal lineage. Moreover, *Myh6* expression was detected, which is expressed in cardiomyocytes (Figure S2B in Supplementary Material).

Four iPSC lines from four different mice formed teratomas in immunodeficient recipients indicating successful reprogramming of the fibroblasts into iPSCs (Figure S2C in Supplementary Material). These features are summarized in Table 1. The iPSCs were also injected subcutaneously into the respective donors of the fibroblasts. In three of the five mice, the autologous iPSCs formed teratomas (Figure 1C). The iPSC lines were also injected in syngeneic (not autologous) C57BL/6J recipients (H2^b^) as well as into MHC-matched but otherwise allogeneic 129Sv mice (H2^b^). Teratomas were observed in about 40% of the syngeneic but none of the allogeneic recipients (Table 1).

**Table 1:** Features of induced pluripotent stem cell (iPSC) lines generated for testing teratoma growth in autologous recipients.

iPSC line	Expression of pluripotency genes (mRNA)	Expression of pluripotency markers (protein)	*In vitro* differentiation (expression of marker genes)	Teratoma in immunodeficient recipients (RAG2^−/−^)	Teratoma in autologous recipient (C57BL/6J)	Teratoma in syngeneic Recipients (C57BL/6J)	Teratoma in major histocompatibility complex-matched allogeneic recipients (129 Sv, H2^b^)
0–3	Yes	Yes	Yes	2/3	Yes	2/4	0/3
1–2	Yes	Yes	Yes	4/4	Yes	2/4	0/3
6–5	Yes	Yes	Yes	0/4^1^	Yes	0/4	0/3
8–7	Yes	Yes	Yes	4/4	No	2/4	0/3
9–2	Yes	Yes	Yes	nt^2^	No	nt	nt

*^1^The iPSC 6–5 cells formed teratomas in two of two RAG2^−/−^γc^−/−^ mice, which lack in addition to B and T cells also NK cells, and were thereby confirmed to be pluripotent*.

*^2^The abbreviation nt indicates not tested*.

**Figure 2:**
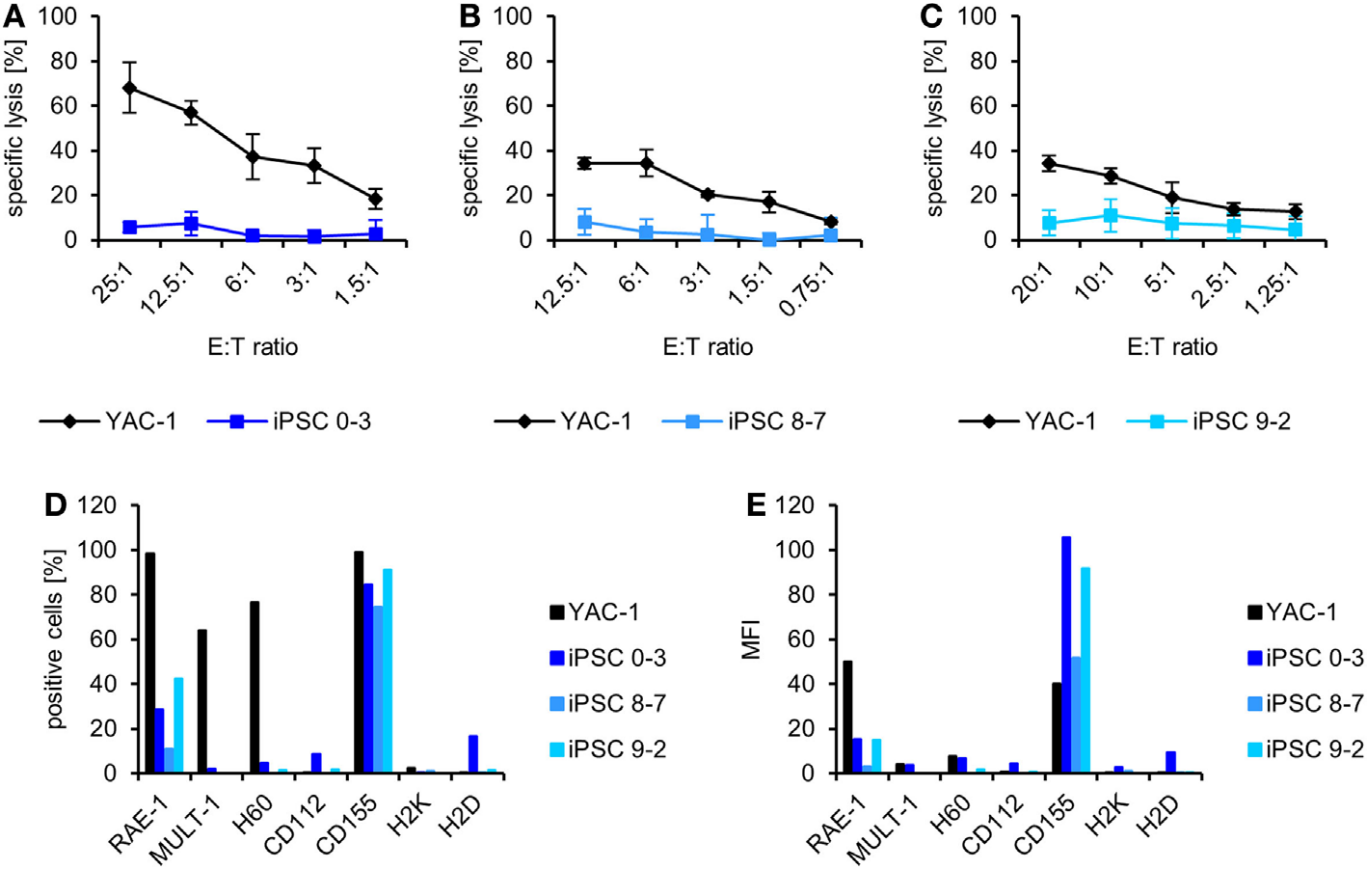
Natural killer (NK) cells of mice injected with autologous induced pluripotent stem cell (iPSCs) do not kill the respective iPSC line *ex vivo*. **(A)** Means and SD of triplets are displayed of the specific lysis of YAC-1 and iPSC 0–3 cells by NK cells derived from the donor mouse of this iPSC line, which had been injected with these cells and developed a teratoma. **(B)** Means and SD of triplets are shown of the specific lysis of YAC-1 and iPSC 8–7 cells by NK cells derived from the donor mouse of this iPSC line, which had been injected with these cells and developed no teratoma. **(C)** Means and SD of triplets are displayed of the specific lysis of YAC-1 and iPSC 9–2 cells by NK cells derived from the donor mouse of this iPSC line, which had been injected with these cells but developed no teratoma. **(D)** In parallel to the ^51^Cr-release assays, shown in panels **(A–C)**, the expression of ligands of activating and inhibitory NK receptors was determined by flow cytometry and the percentage of cells expressing the indicated ligands is shown. **(E)** The expression intensity of the ligands is displayed as mean fluorescence intensity.

From three of these mice injected with autologous iPSCs [two without (8–7 and 9–2) and one with a teratoma (0–3)], we obtained NK cells at the end of the experiment and tested their activity against the autologous iPSCs and the control cell line YAC-1 directly *ex vivo* without any further *in vitro* stimulation. The NK cells of all mice killed YAC-1 targets but failed to lyse the respective iPSC lines (Figures 2A–C) despite expressing ligands of activating NK receptors, including the NKG2D ligand RAE-1 and the DNAM-1 ligand CD155 and despite being largely negative for MHC class I molecules, which serve as ligands for inhibitory NK receptors of the Ly49 family (Figures 2D,E).

### Efficient Killing of PSCs Requires Cytokine-Activated NK Cells

We have previously shown that IL-2-activated NK cells efficiently kill PSCs, including iPSCs, ESCs, and maGSCs (44). However, the NK cells from the C57BL/6 mice, which had been injected with autologous iPSCs in this study, failed to kill the iPSCs, irrespective of whether they developed a teratoma or not. Therefore, we next compared the efficacy of resting and IL-2-activated NK cells against several PSC lines. These included an iPSC line from a C57BL/6 mouse and an iPSC line from a 129Sv mouse, which were generated similarly by transduction of fibroblasts with the STEMCCA virus. We also included a newly established ESC line from a 129Sv embryo (ESC BTL1) (Figure S3 in Supplementary Material) and the long-established ESC line MPI-II also derived from a 129Sv embryo (31). Two maGSC lines, one from a C57BL/6 and one from a 129Sv mouse, which have been previously described (10, 44), were also included in the experiments. The pluripotency of the new stem cell lines iPSC 129Sv, iPSC C57BL/6, and ESC BTL1 cells was demonstrated by their ability to differentiate *in vivo* into derivatives of the three germ layers in teratoma assays (Figure S4 in Supplementary Material).

**Figure 3:**
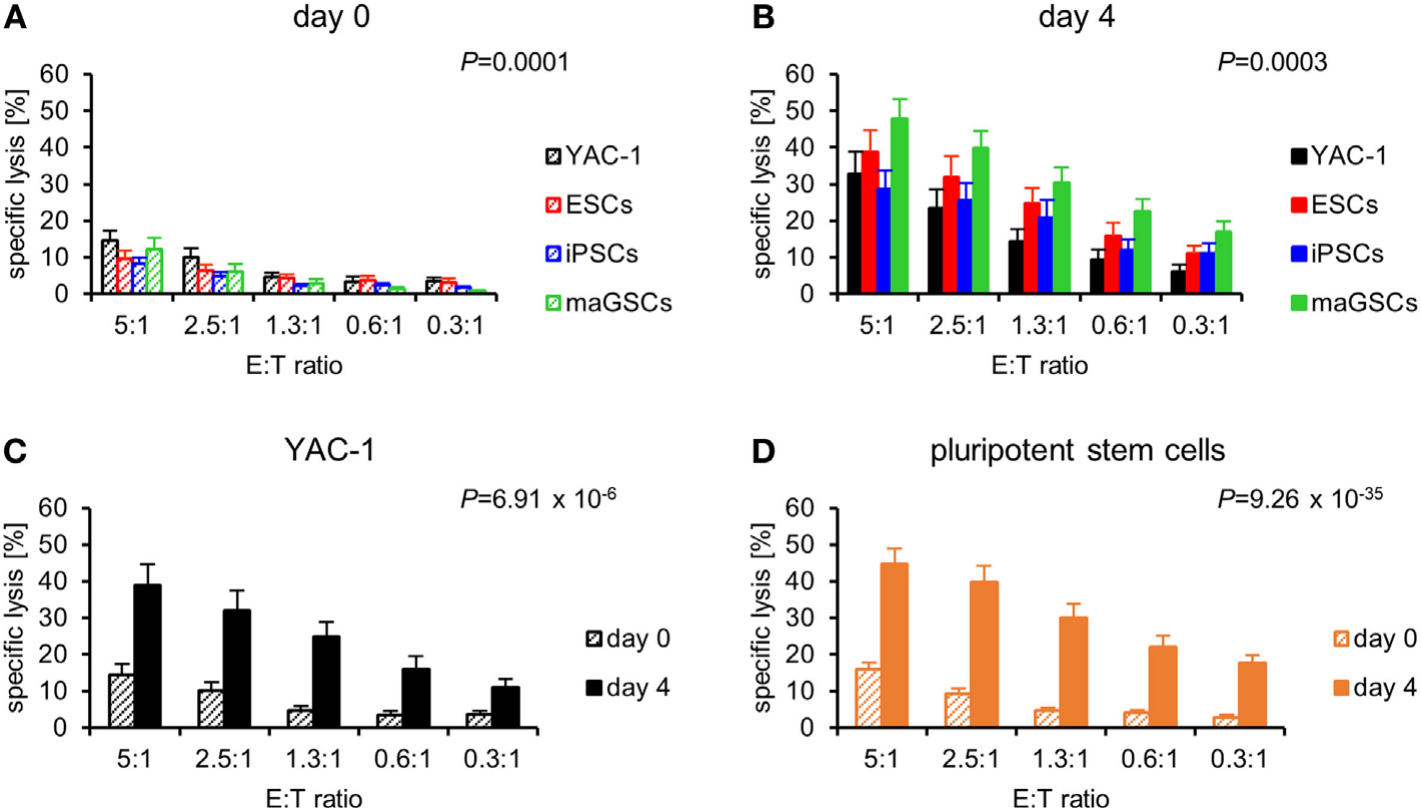
Pluripotent stem cells (PSCs) are more susceptible to interleukin (IL)-2-activated natural killer (NK) cells than resting NK cells. **(A)** A summary of means and SEM of specific lysis of YAC-1 cells (*n* = 16), embryonic stem cells (ESCs) (*n* = 14), induced pluripotent stem cell (iPSCs) (*n* = 18), and multipotent adult germline stem cell (maGSCs) (*n* = 16) by freshly purified NK cells (day 0) is shown as determined by ^51^Cr-release assays. **(B)** A summary of means and SEM of specific lysis of YAC-1 cells (*n* = 12), ESCs (*n* = 12), iPSCs (*n* = 12), and maGSCs (*n* = 12) by IL-2-activated NK cells (day 4) is shown as determined by ^51^Cr-release assays. The cytotoxic activity of resting (day 0) and IL-2-activated NK cells (day 4) has been compared for **(C)** YAC-1 cells and **(D)** all PSCs together. *P*-values for the comparisons (2-way-analyses of variance adjusted for E:T ratios) are indicated.

We purified NK cells from C57BL/6 mice and either stimulated them for 4 days *in vitro* with IL-2 or used them directly at day 0 as effector cells in ^51^Cr-release assays against the PSCs and YAC-1 targets. Resting NK cells (day 0) killed the target cells with different efficacy (*P* = 0.0001, ANOVA) (Figure 3A). The YAC-1 control target cells were more susceptible to resting NK cells than iPSCs (*P* = 9.47 × 10*^−^*^6^) and maGSCs (*P* = 0.0096) and there was a similar trend for ESCs (*P* = 0.0766, Bonferroni *post hoc* test). In a comparison of the PSC types only, a trend for variation in their susceptibility to resting NK cells was found (*P* = 0.0575). Similarly, the four target types varied in killing by IL-2-activated NK cells at day 4 (*P* = 0.0003) (Figure 3B). The YAC-1 cells were less susceptible to activated NK cells than maGSCs (*P* = 0.0010, Bonferroni *post hoc* test). The susceptibility of the PSC types only also varied (*P* = 0.0007), and the *post hoc* test indicated a higher killing of maGSCs than iPSCs (*P* = 0.0006) and ESCs (*P* = 0.0389). Thus, maGSCs were more susceptible to resting and activated NK cells than the other PSC types.

The comparison of the susceptibility of the targets to resting and cytokine-activated NK cells indicated that the YAC-1 cells were more susceptible to IL-2-activated NK cells (*P* = 6.91 × 10*^−^*^6^) (Figure 3C), similarly also the PSCs were overall significantly better lysed by IL-2-activated than by resting NK cells (*P* = 9.26 × 10*^−^*^35^) (Figure 3D). This difference was confirmed for all three types of PSCs in separate analyses (Figure S5 in Supplementary Material).

### The Cytotoxic Activity of Resting NK Cells against PSCs Depends on the Activating NK Receptor NKG2D

**Figure 4:**
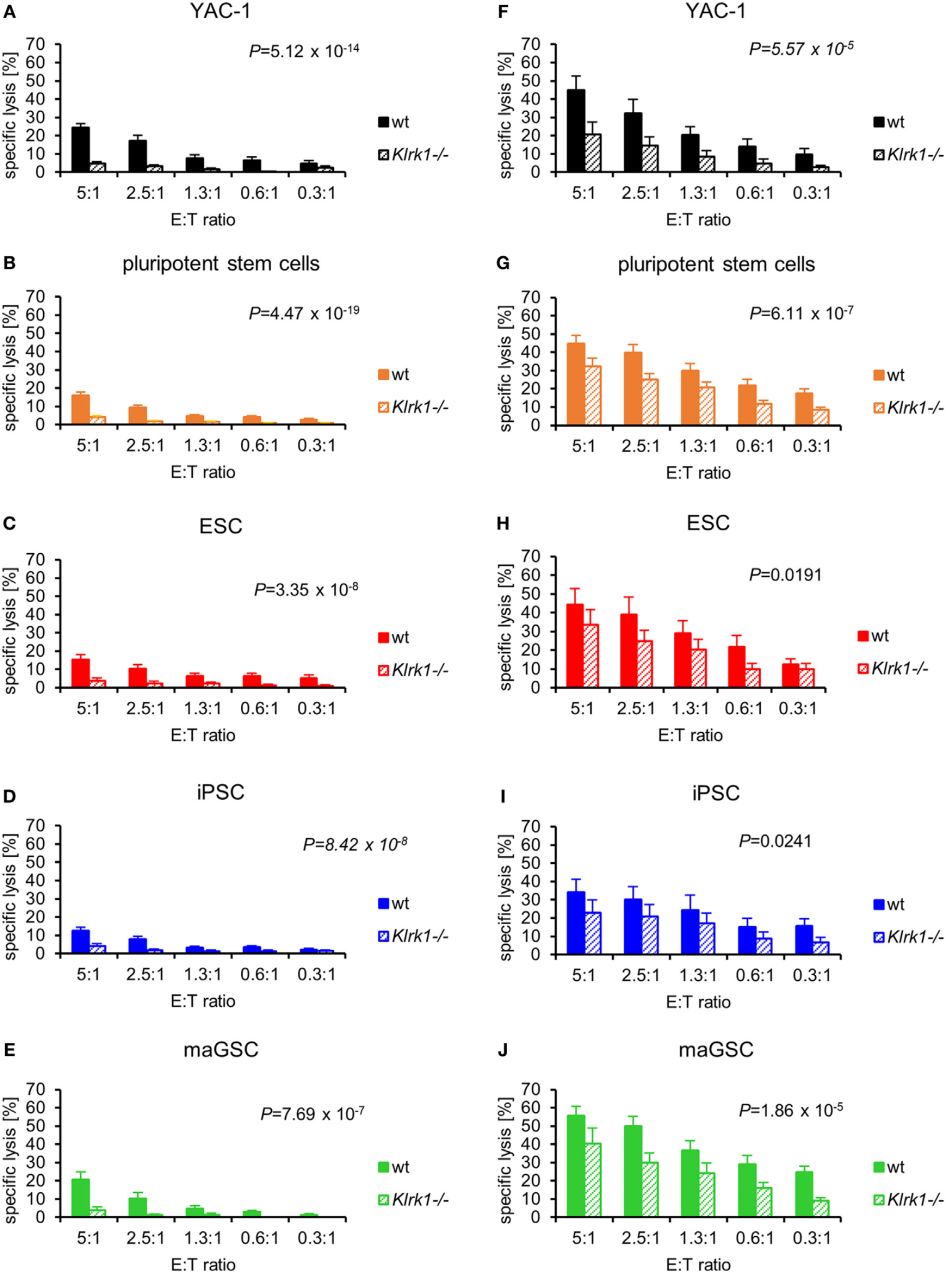
Pluripotent stem cells (PSCs) are largely resistant against resting NKG2D-deficient natural killer (NK) cells, whereas the cytotoxic activity of interleukin-2-activated NK cells depends only partly on NKG2D. The cytotoxic activity of resting wild-type (wt) and NKG2D-deficient (*Klrk1^−^*^/^*^−^*) NK cells has been compared for **(A)** YAC-1 cells (wt: *n* = 8, *Klrk1^−^*^/^*^−^*: *n* = 8), **(B)** all PSCs together (wt: *n* = 24, *Klrk1^−^*^/^*^−^*: *n* = 24), **(C)** embryonic stem cells (ESCs) (wt: *n* = 7, *Klrk1^−^*^/^*^−^*: *n* = 7), **(D)** induced pluripotent stem cells (iPSCs) (wt: *n* = 9, *Klrk1^−^*^/^*^−^*: *n* = 9), and **(E)** multipotent adult germline stem cell (maGSCs) (wt: *n* = 8, *Klrk1^−^*^/^*^−^*: *n* = 8). The cytotoxic activity of IL-2-activated wt and *Klrk1^−^*^/^*^−^* NK cells has been compared for **(F)** YAC-1 cells (wt: *n* = 6, *Klrk1^−^*^/^*^−^*: *n* = 6), **(G)** all PSCs together (wt: *n* = 18, *Klrk1^−^*^/^*^−^*: *n* = 18), **(H)** ESCs (wt: *n* = 6, *Klrk1^−^*^/^*^−^*: *n* = 6), **(I)** iPSCs (wt: *n* = 6, *Klrk1^−^*^/^*^−^*: *n* = 6), and **(J)** maGSCs (wt: *n* = 6, *Klrk1^−^*^/^*^−^*: *n* = 6). *P*-values for the comparisons (2-way-analyses of variance adjusted for E:T ratios) are indicated.

In inhibition experiments with recombinant NKG2D-Fc proteins, we have found previously that the killing of PSCs by IL-2-activated NK cells partly depends on the activating NK receptor NKG2D (31, 44). To verify this result and to compare resting and IL-2-activated NK cells, we used NKG2D-deficient mice (*Klrk1^−^*^/^*^−^*) as NK cell donors in addition to the wild-type mice. Resting NKG2D-deficient NK cells were significantly impaired in killing of YAC-1 target cells (Figure 4A). The PSC lines were hardly killed and overall more resistant to NKG2D-deficient than to wild-type NK cells (*P* = 4.47 × 10*^−^*^19^) (Figure 4B). The separate analysis of the three PSC types revealed that all types were significantly more resistant to NKG2D-deficient than to wild-type NK cells (Figures 4C–E).

### The Cytotoxic Activity of Cytokine-Activated NK Cells against PSCs Depends Partly on NKG2D

The cytotoxicity of IL-2-activated NK cells against YAC-1 cells was largely dependent on NKG2D (*P* = 5.57 × 10*^−^*^5^) (Figure 4F). Overall, also the lysis of PSCs by NKG2D-deficient NK cells was reduced (*P* = 6.11 × 10*^−7^*) (Figure 4G). Although the IL-2-activated NK cells were less dependent on NKG2D than resting NK cells, the killing of ESCs, iPSCs, and maGSCs by NKG2D-deficient NK cells was lower than the killing by wild-type IL-2-activated NK cells (Figures 4H–J).

### Individual PSC Lines Vary in Their Susceptibility to NK Cells Independent of the Stem Cell Type

Separate analyses of the single PSC lines for their susceptibility to resting and IL-2-activated wild-type and NKG2D-deficient NK cells are shown in the Figure S6 in Supplementary Material. The six PSC lines included in the analysis varied in their susceptibility to IL-2-activated NK cells (*P* = 0.0010, ANOVA), but to resting NK cells only with borderline significance (*P* = 0.0634, ANOVA). The maGSC 129Sv cells were killed better by IL-2-activated NK cells than ESC MPI-II (*P* = 0.0078) and iPSC 129Sv cells (*P* = 0.0092, Bonferroni *post hoc* test). All cell lines were more susceptible to IL-2-activated NK cells than resting NK cells and the cytotoxic activity of resting NK cells toward all PSC lines was NKG2D-dependent. However, only for ESC BTL1, maGSC 129Sv, and maGSC C57BL/6 cells the killing by IL-2-activated NK cells was significantly impaired by NKG2D deficiency (Figure S6 in Supplementary Material).

### The PSC Lines Express NKG2D Ligands

**Figure 5:**
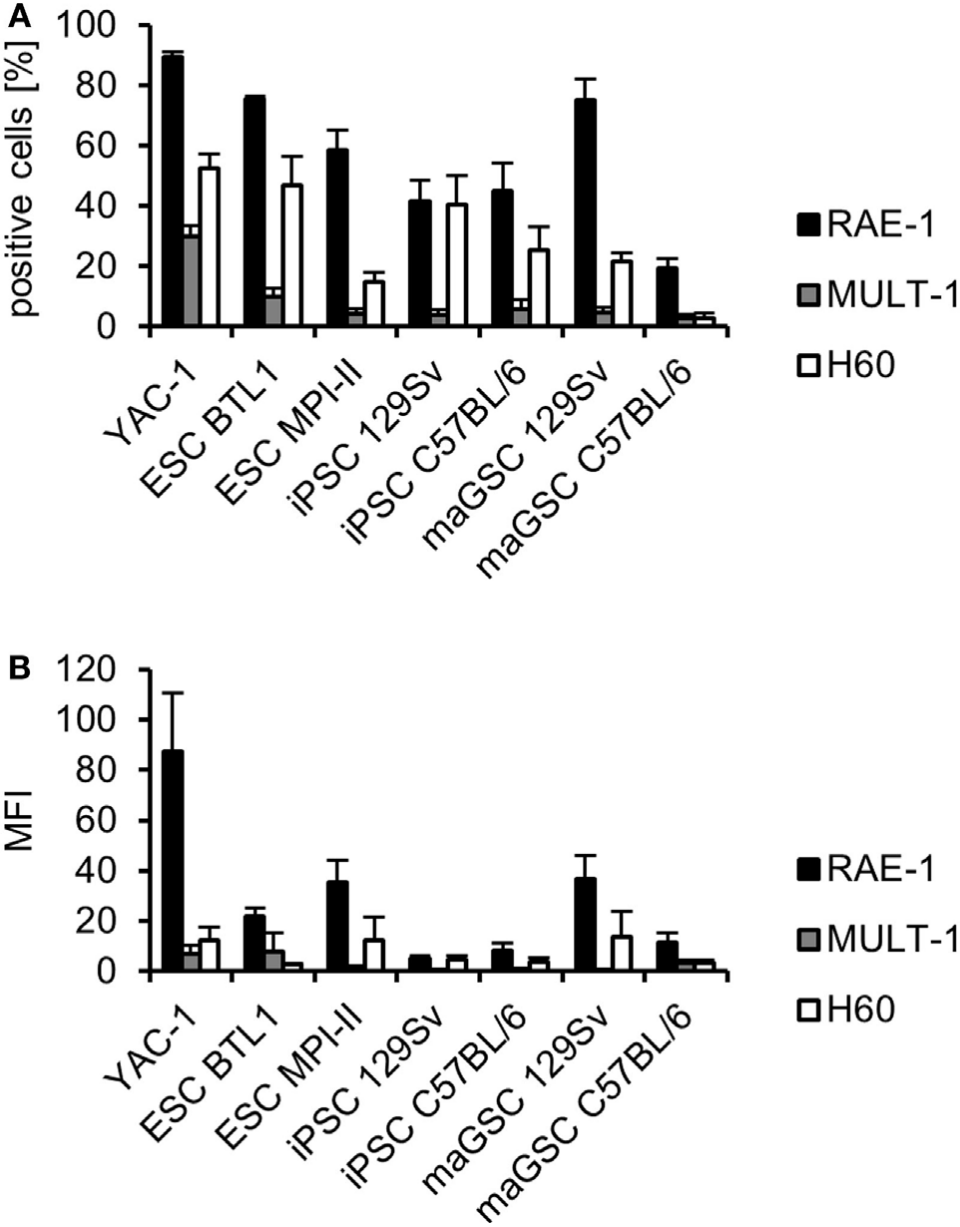
Analysis of the expression of NKG2D ligands on target cells. **(A)** The percentage of cells expressing the indicated NKG2D receptor ligands (mean plus SEM) is shown as determined by flow cytometry in 3–13 individual experiments [YAC-1: *n* = 13, embryonic stem cell (ESC) BTL1: *n* = 3, ESC MPI-II: *n* = 7, induced pluripotent stem cell (iPSC) 129Sv: *n* = 5, iPSC C57BL/6: *n* = 7, multipotent adult germline stem cell (maGSC) 129Sv: *n* = 6, maGSC C57BL/6: *n* = 7]. **(B)** The mean fluorescence intensity has been determined in parallel and is displayed plus SEM.

The PSC lines expressed the NKG2D ligands RAE-1, MULT-1, and H60 in variable amounts as determined by flow cytometry in parallel to the cytotoxicity assays (Figure 5). On average more than 50% of the ESC BTL1, ESC MPI-II, and maGSC 129Sv cells expressed RAE-1 (Figure 5A) and the MFI of RAE-1 (21.6, 36.6, and 36.6) was higher than for some other PSC lines, such as iPSC 129Sv (4.9) (Figure 5B). Taking into account the proportion of positive cells and the MFI, these data are summarized in Table 2. The expression of MHC class I molecules (H2D^b^ and H2K^b^) has also been determined and the newly established PSC lines were negative similarly as the old lines (Table 2), which had been tested also in previous studies (31, 44, 46). This expression pattern fits well to the observation that the killing of these cell lines by IL-2-activated NK cells was in part dependent on NKG2D. However, the lowest expression of NKG2D ligands was observed on the maGSC C57BL/6 cells but their killing by IL-2-activated NKG2D-deficient NK cells was also clearly reduced. Thus, further factors appear to contribute to these variations and lack of inhibition by inhibitory NK receptors, which bind to MHC class I molecules is presumably one of them.

**Table 2:** Summary of the expression of NKG2D ligands and major histocompatibility complex class I molecules on pluripotent stem cells and YAC-1 cells.

Cell line	RAE-1	MULT-1	H60	H2D^b^	H2K^b^
YAC-1	+++^1^	++	++	nt^2^	nt
ESC BTL1	+++	+		–	–
ESC MPI-II	+++	–		–	–
iPSC 129Sv	++	–		–	–
iPSC C57BL/6	++	–		–	–
maGSC 129Sv	+++	–		–	–
maGSC C57BL/6	++	–	–	–	–

*^1^The symbol +++ indicates that on average the log10(%-positive cells × mean fluorescence intensity) is ≥3, ++ is <3 and ≥2, + is <2 and ≥1, and − is <1*.

*^2^The abbreviation nt indicates not tested. YAC-1 cells carry the H2^a^ haplotype*.

## Discussion

It is well-known that ESCs can form teratomas not only in immunodeficient but also in immunocompetent syngeneic hosts (29, 31). Therefore, it is reasonable to expect that iPSCs will form teratomas upon transplantation into autologous recipients, although this has previously not been demonstrated to our knowledge. In this study, we have shown that murine iPSCs derived from fibroblasts indeed can form teratomas in the individual mice from which the fibroblasts have been taken. Notably, teratomas are regularly formed in syngeneic immunocompetent recipients although syngeneic NK cells can attack PSCs (31, 44). When we compared the teratoma growth upon subcutaneous injections of ESC MPI-II cells into immunocompetent syngeneic 129Sv mice and immunodeficient SCID mice, which have no B and T cells, and SCID/beige mice, which in addition lack also functional NK cells, we found that the teratoma growth was similarly impaired in 129Sv and SCID mice compared to SCID/beige mice (22). Moreover, depletion of NK cells in SCID mice supported the early outgrowth of teratomas after injection of PSCs (44). These results demonstrated that NK cells indeed impair teratoma growth but are not sufficient to completely prevent tumors if high enough numbers of PSCs are injected. Activation of NK cells *in vivo* by poly I:C further reduced the teratoma frequency and size in SCID but not in SCID/beige recipients (44), suggesting that the activation status of NK cells is important for the outcome. Importantly, *in vitro*-differentiated cells have been shown to acquire resistance to NK cells (44), and therefore, PSCs are expected to escape finally the NK cell-mediated rejection by differentiation into other cell types which then form the teratoma.

The autologous transplantations of iPSCs performed in this study did not lead to a significant cytotoxic activity of NK cells against the autologous iPSCs. This was observed similarly in one mouse that developed a teratoma and in two recipients without a teratoma. We have recently shown that NK cells impair the outgrowth of teratomas from maGSCs not only in the subcutaneous tissue but also after transplantation into a therapeutically relevant tissue such as the myocardium (45). In these experiments, it turned out that the operation procedure, which included an open chests surgery, was substantially increasing the cytotoxic activity of NK cells up to 4 weeks after transplantation (45). The subcutaneous injections of the autologous iPSCs performed in the experiments reported here, produced presumably less inflammatory responses than a major surgery and consequently did not activate NK cells against the iPSCs at least at the time point when the mice were sacrificed. However, individual differences might exist since NK cells from mouse 0–3 showed a higher cytotoxic activity against YAC-1 control target cells than the NK cells of the other two recipients of autologous iPSCs (8–7, 9–2). NK cells of these two mice killed YAC-1 cells to a similar extent as NK cells from untreated control C57BL/6 mice that we have analyzed previously (45).

Taking the importance of the activation status of NK cells into account, we now directly compared the cytotoxic activity of resting and IL-2-activated NK cells against PSCs. As in our previous studies (31, 44), all PSC lines, including ESCs, iPSCs, and maGSCs, were targets for IL-2-activated NK cells. However, they were much less attacked by resting than activated NK cells. The maGSCs appeared to be killed even better than ESCs and iPSCs by activated NK cells but differences of individual PSC lines might be more important for this finding than principal differences between the PSC types. The finding that murine PSCs are good targets for cytokine-activated NK cells but are killed less by resting NK cells likely explains why some early studies reported resistance of ESCs to NK cells (40, 41) while we and others found them to be susceptible to cytokine-activated NK cells (31, 42, 44). The result is also in agreement with our findings on human iPSCs, which were killed by allogeneic and syngeneic IL-2-activated NK cells but much less by resting NK cells (39), and with results reported by others for human cardiac-derived stem/progenitor cells (55).

Murine PSCs do not express MHC class I molecules at least at a level that is readily detectable by flow cytometry (31, 44, 46). MHC class I molecules function as ligands mainly for inhibitory NK receptors of the Ly49 family in mice (35). Cells that lack MHC class I molecules cannot inhibit NK cell activity and can, therefore, become targets of NK cells (56). Importantly, the activity of NK cells is regulated by the balance of inhibitory and activating signals received *via* inhibitory and activating NK receptors (37). One of the most important activating NK receptor is NKG2D that interacts with a group of stress-inducible ligands including the RAE-1 and H60 families and MULT-1 in the mouse (36, 57). We and others have shown that murine ESCs are among the few cell types that constitutively express NKG2D ligands (31, 42). In addition to ESCs, also iPSCs and maGSCs express variable patterns of NKG2D ligands mainly of the RAE-1 family and ligands of further activating NK receptors including the DNAM-1 ligands CD112 and CD155 (44). The system of NKG2D and NKG2D ligands has been shown to be highly important for tumor immunity (58). It also affects the outcome of hematopoietic stem cell transplantation (59–62) and solid organ transplantation (63, 64). NKG2D has also been reported to be involved in the rejection of neural progenitor cells by NK cells (65). In inhibition experiments with a recombinant NKG2D protein, we have previously shown that the killing of murine ESCs (31) and other PSCs (44) by IL-2-activated NK cells is partly dependent on NKG2D. Here, we have confirmed this finding by comparing the activity of wild-type and genetically NKG2D-deficient NK cells. Notably, the killing of some individual PSC lines appeared to depend little on NKG2D while killing of others was clearly affected by NKG2D-deficiency. Thus, the only partial inhibition of killing by recombinant NKG2D, which we observed in previous experiments, was not due to an incomplete inhibition of NKG2D activity and other activating NK receptors than NKG2D must contribute to the recognition of PSCs by activated NK cells. DNAM-1 is an obvious candidate among others because especially the ligand CD155 is prominently expressed on PSCs including the autologous iPSCs used in this study. Moreover, we have recently shown that the killing of human iPSCs by NK cells is mediated in part by DNAM-1 (39). The killing of human iPSCs was not dependent on NKG2D with exception of one combination of a specific iPSC line and an individual NK cell donor (39). Thus, individual variations in NK receptor–ligand interaction need to be considered for their implications on human transplantations.

This potential individual variation in recognitions of PSCs by NK cells is further emphasized by results obtained with human ESCs that have been engineered not to express β2-microglobulin (66). In contrast to murine PSCs, human ESCs as well as iPSCs do express significant amounts of MHC class I molecules (39, 47). The β2-microglobulin and, therefore, also MHC class I-deficient human ESCs were killed more efficiently by NK cells than wild-type ESCs (66). This demonstrates the relevance of NK cell inhibition *via* human MHC class I molecules recognized by inhibitory KIRs on human NK cells. Recognition of human MHC class I molecules by KIRs shows a considerable variability (34) and the activity of NK cells toward allogeneic PSCs and their differentiation products can, therefore, be expected to vary depending on the MHC polymorphism of the PSCs and the KIR polymorphism of the recipients.

Notably, the low cytotoxic activity of resting NK cells against PSCs, which we observed, was almost completely depend on NKG2D in all investigated PSC lines. Therefore, any *in vivo* activity of NK cells against PSCs under non-inflammatory conditions must be expected to depend largely on the recognition of NKG2D ligands by NKG2D at least in murine transplantation models. Subcutaneous injections of PSCs presumably cause little inflammatory responses compared to, e.g., intramyocardial injections by open chest surgery. Nonetheless, we observed measurable effects of NK cells on the teratoma growth kinetics after injection of PSCs in both models (44, 45). Thus, also the low largely NKG2D-dependent cytotoxic activity of resting NK cells against PSCs appears to be relevant *in vivo*. It might be highly important if PSCs would be present in trace amounts among *in vitro*-differentiated cells intended for grafting. If NK cells contribute to a fail-safe system against the tumorigenicity of PSCs after transplantation (67), this system might largely rely on NKG2D.

As a caveat, it should be mentioned that the initial step or even steps of differentiation of PSCs *in vivo* must not immediately remove the risk of tumorigenicity associated with PSCs but it could alter the recognition of the differentiated cells by the immune system and particularly NK cells. When we transplanted the murine ESC line MPI-II into rats immunosuppressed with cyclosporine A, no teratomas occurred likely due to the rejection of the ESCs by NK cells (31) which are not suppressed by cyclosporine A in their activity against PSCs (45). However, when the same numbers of neuronal cells with a purity of more than 95% that were obtained from these ESCs by a directed *in vitro* differentiation protocol were injected, we observed teratomas in 61% of the recipients (31). Thus, a cell type in the differentiation culture that differed from the original ESCs by resistance to NK cells but that retained pluripotency presumably caused the tumors in these experiments. Therefore, it is important to carefully investigate the individual immunological properties of any graft that is evaluated in preclinical animal models or in clinical studies. Differentiation of PSCs into progenitor cells with a limited differentiation potential, e.g., neural progenitor cells, might be a strategy to significantly reduce the risk of tumorigenicity (68).

## Conclusion

Transplantation of autologous iPSCs can lead to teratomas as formally demonstrated here in a murine model to our knowledge for the first time. Transplantation of grafts derived from autologous iPSCs into immunocompetent recipients as well as grafts derived from allogeneic PSCs into immunosuppressed or immunomodulated recipients has the unavoidable risk of tumorigenicity if undifferentiated or not sufficiently differentiated cells are present in the grafts. The cytotoxic activity of NK cells against PSCs might reduce this risk although NK cells need to be activated by cytokines to exhibit a high cytotoxicity against PSCs. The IL-2-activated NK cells killed PSCs in a partly NKG2D-dependent manner while the low killing of PSCs by resting NK cells was almost completely dependent on NKG2D. Thus, any activity of NK cells against PSCs present in grafts transplanted under non-inflammatory conditions, which fail to produce NK cell-activating cytokines, will presumably depend largely on NKG2D at least in murine models. The analysis of the activity of cytokine-activated NK cells against PSCs and of the receptors involved might allow for the development of immunomodulatory protocols, which stimulate NK cells to function efficiently as safeguard against undifferentiated PSCs in PSC-derived grafts.

## Ethics Statement

All animal experiments had been approved by the local government (Niedersächsisches Landesamt für Verbraucherschutz und Lebensmittelsicherheit) and were carried out in compliance with German and EU legislation (Directive 2010/63/EU).

## Author Contributions

RD and KG designed the study; CG, DH, JN, SM, LE, AS, and RD acquired data; JN, CT, WP, BP, AM, and KG provided important reagents, CG, DH, JN, SM, AS, AM, KG, and RD analyzed and interpreted data; RD wrote the manuscript; all authors approved the final version of the manuscript.

## Conflict of Interest Statement

The authors declare that the research was conducted in the absence of any commercial or financial relationships that could be construed as a potential conflict of interest.
