# Supplementary material for: Efficient Killing of Murine Pluripotent Stem Cells by Natural Killer (NK) Cells Requires Activation by Cytokines and Partly Depends on the Activating NK Receptor NKG2D
Source: Front Immunol. 2017 Jul 26;8:870. doi: 10.3389/fimmu.2017.00870 (PMC5582315; doi:10.3389/fimmu.2017.00870)
Supplement: Supplementary file 1 [file Presentation_1.pdf]

Supplementary Materials

Efficient killing of murine pluripotent stem cells by natural killer (NK) cells requires activation by cytokines and partly depends on the activating NK receptor NKG2D

Carina Gröschel, Daniela Hübscher, Jessica Nolte, Sebastian Monecke, André Sasse, Leslie Elsner, Walter Paulus, Claudia Trenkwalder, Bojan Polic, Ahmed Mansouri, Kaomei Guan, Ralf Dressel*

*** Correspondence:** Ralf Dressel: rdresse@gwdg.de

1 Supplementary Figures and Tables

- Supplementary Tables 1 to 3
- Supplementary Figures 1 to 6

1.1 Supplementary Tables

Supplementary Table 1. Antibodies for immunofluorescence (IF) and immunoblotting (IB)

Antigen	Isotype	Clone	Supplier	Label	Assay	Dilution
KLF4	rabbit IgG	polyclonal (ab34814)	Abcam, Cambridge, United Kingdom	-	IB	1:1000
LIN28	goat IgG	polyclonal (YFC01)	R&D Systems, Wiesbaden, Germany	-	IF	1:100
NANOG	goat IgG	polyclonal (AF2729)	R&D Systems, Wiesbaden, Germany	-	IF	1:200
OCT4	rabbit IgG	polyclonal (ab18976)	Abcam, Cambridge, United Kingdom	-	IB	1:1000
SALL4	rabbit IgG	polyclonal (ab29112)	Abcam, Cambridge, United Kingdom	-	IB	1:1000
SOX2	rabbit IgG	polyclonal (ab59776)	Abcam, Cambridge, United Kingdom	-	IB	1:1000
SSEA1	mouse IgM	MC480	Developmental Studies Hybridoma Bank (DSHB), Iowa City, Iowa, USA	-	IF	undiluted hybridoma supernatant
α-Tubulin	mouse IgG_1	B-5-1-2	Sigma, Darmstadt, Germany	-	IB	1:10000
ZFP206	rabbit IgG	polyclonal	kindly provided by L.W. Stanton, Singapore	-	IB	1:2000
goat IgG	monkey anti-goat IgG	polyclonal (705-166-147)	Jackson Laboratories, via Dianova, Hamburg, Germany	Cy3	IF	1:600
mouse IgM	Goat anti-mouse IgG+IgM	polyclonal (115-165-068)	Jackson Laboratories, via Dianova, Hamburg, Germany	Cy3	IF	1:600

mouse IgG	Horse anti-mouse IgG	polyclonal (#7076)	Cell Signaling Technology, Danvers, Massachusetts, USA	HRP	IB	1:10000

Supplementary Table 2. Primers used for RT-PCR or qPCR

Gene	Sequence 5'-3'	Assay
Afp	F: CCC ACC CTT CCA GTT TCC<br>R: TAC TGA GCA GCC AAG G	RT-PCR
Flk1	F: CCT ACC CCA CAC ATT ACA TGG<br>R: TTT TCC TGG GCA CCT TCT ATT	RT-PCR
Gapdh	F: GCA GTG GCA AAG TGG AGA TT<br>R: TCT CCA TGG TGG TGA AGA CA	RT-PCR
Hprt	F: AGC CCC AAA ATG GTT AAG GTT GC<br>R: TTG CAG ATT CAA CTT GCG CTC AT	qPCR
Klf4	F: TCA GGT ACC CCT CTC TCT TCT TTC<br>R: CGC TTC ATG TGA GAG AGT TCC T	qPCR
Lin28	F: TCC TCC TGT GTC TCC CAT TC<br>R: AGA GTG AGG CCC TGT CTC AA	RT-PCR
Mash1 (Ascl1)	F: CTC GTC CTC TCC GGA ACT GAT G<br>R: CGA CAG GAC GCC GCG CTG AAA G	RT-PCR
Myh6	F: CTG CTG GAG AGG TTA TTC CTC G<br>R: GGA AGA GTG AGC GGC GCA TCA AGG	RT-PCR
Nanog	F: AGG GTC TGC TAC TGA GAT GCT CTG<br>R: CAA CCA CTG GTT TTT CTG CCA CCG	RT-PCR
Nanog	F: TTA CAA GGG TCT GCT ACT GAG ATG<br>R: CAG GAC TTG AGA GCT TTT GTT TG	qPCR
Oct4	F: GGC GTT CTC TTT GGA AAG GTG TTC<br>R: CTC GAA CCA CAT CCT TCT CT	RT-PCR
Oct4	F: CGG AAG AGA AAG CGA ACT AGC<br>R: GCC TCA TAC TCT TCT CGT TGG	qPCR
Sox2	F: GGC GGC AAC CAG AAG AAC AG<br>R: GCT TGG CCT CG TCG ATG AAC	RT-PCR
Zfp206	F: GAG AGG AGG TGG TAC AGC TAT TG<br>R: AGG TGG AGG TAA CTC ATT CAG TG	qPCR

Supplementary Table 3. Antibodies and isotype controls used for flow cytometry

**Antigen**	**Isotype**	**Clone**	**Label**	**Supplier**
CD3	rat IgG_{2b}	17A2	FITC	BioLegend, Fell, Germany
CD49b	rat IgM	DX5	PE	BioLegend, Fell, Germany
CD112	rat IgG_{2a}	502-57	-	Santa Cruz, Heidelberg, Germany
CD155	rat IgG_{2a}	TX56	-	BioLegend, Fell, Germany
CD314 (NKG2D)	mouse IgG_1	149810	PE	R&D Systems, Wiesbaden, Germany
$H2K^b$	mouse IgG_{2a}	AF6-885	PE	BioLegend, Fell, Germany
$H2D^b$	mouse IgG_{2b}	KH95	PE	BioLegend, Fell, Germany
H60	rat IgG_{2a}	205326	-	R&D Systems, Wiesbaden, Germany
MULT-1	rat IgG_{2a}	205326		R&D Systems, Wiesbaden, Germany
RAE-1	rat IgG_{2a}	186107	-	R&D Systems, Wiesbaden, Germany
rat IgG	goat IgG	polyclonal (112-095-062)	FITC	Jackson Laboratories, via Dianova, Hamburg, Germany
-	mouse IgG_1	MOPC-21	PE	BioLegend, Fell, Germany
-	mouse IgG_{2a}	MOPC-173	PE	BioLegend, Fell, Germany
-	mouse IgG_{2b}	MPC-11	PE	BioLegend, Fell, Germany
-	rat IgG_{2b}	RTK4530	FITC	BioLegend, Fell, Germany
-	rat IgM	RTK2118	PE	BioLegend, Fell, Germany

The following abbreviations are used: FITC, fluorescein isothiocyanate, and PE, phycoerythrin.

1.2 Supplementary Figures

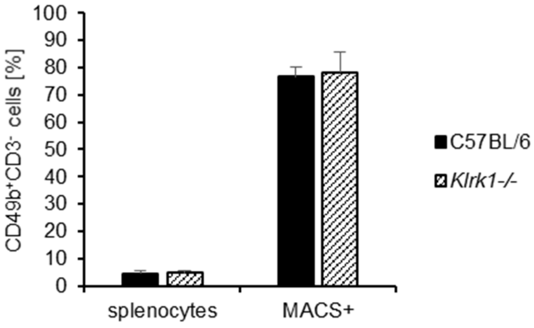

Supplementary Figure 1. The average percentage and SD of CD49b$^+$CD3$^-$ NK cells among splenocytes (C57BL/6: n=26 and *Klrk1-/-*: n=25) and MACS-purified cells (MACS+, n=10) of C57BL/6 and *Klrk1*$^{-/-}$ mice is shown. Splenocytes of two to three mice were used for one MACS separation.

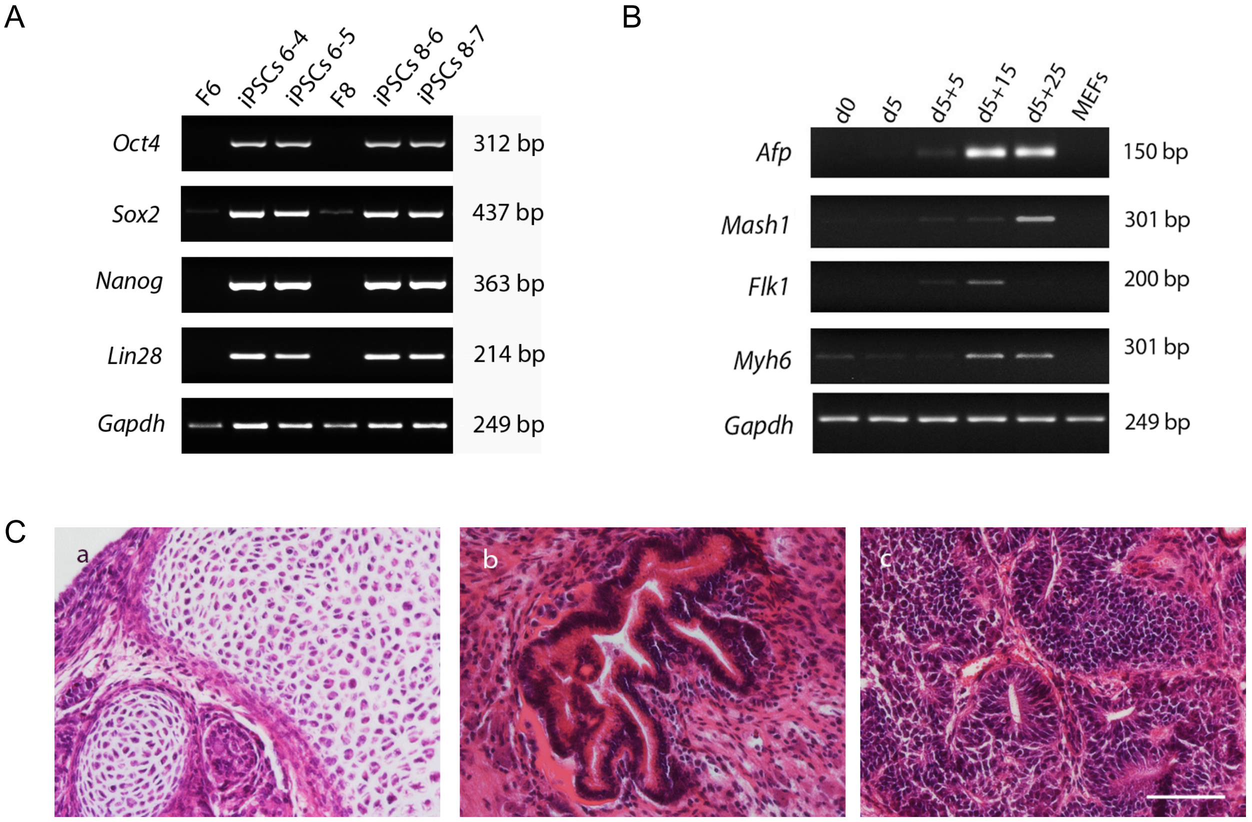

Supplementary Figure 2. The iPSC lines used for autologous transplantation are pluripotent. (A) The expression of pluripotency marker genes (*Oct4*, *Sox2*, *Nanog*, and *Lin28*) and the housekeeping gene *Gapdh* was determined by RT-PCR in fibroblasts and iPSC clones derived from these fibroblasts. This is exemplified here for the fibroblasts F6 and F8 of two donor mice and in two iPSC clones derived from these fibroblasts (6-4, 6-5 and 8-6, 8-7). (**B**) The iPSCs (d0) were differentiated in hanging drops and in suspension for 5 days (d5) and subsequently cultured on 0.1% gelatin-coated dishes for further 5, 15, or 25 days (d5+5, d5+15, d5+25). The expression of marker genes for endoderm (*Afp*), ectoderm (*Mash1*), and mesoderm (*Flk*) was analyzed by RT-PCR as illustrated here for the iPSC line 0-3. Expression of alpha-Mhc (*Myh6*) indicates a differentiation into cardiomyocytes. *Gapdh* was amplified as housekeeping gene and MEFs served as negative control for the marker genes. (**C**) Cells of the iPSC lines were subcutaneously injected into immunodeficient RAG2$^{-/-}$ mice and teratomas were obtained after 35 to 91 days. For iPSC line 1-2, the mesodermal differentiation into cartilage (a), endodermal differentiation into intestinal epithelium (b), and ectodermal differentiation into neural rosettes (c) is exemplified here. The scale bar indicates 100 µm.

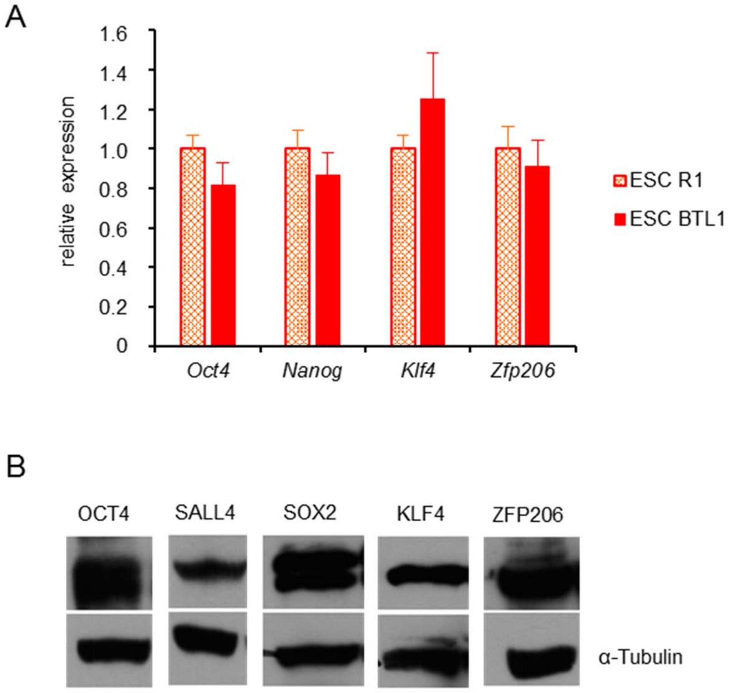

Supplementary Figure 3. The newly generated ESC line BTL1 expresses pluripotency markers. (A) The expression of pluripotency marker genes (*Oct4*, *Nano*g, *Klf1*, and *Zfp206*) was determined in parallel to the housekeeping gene *Hprt* by qPCR in the ESC line BTL1. The mean relative expression of two biological replicates is shown compared to the long established ESC line R1. (**B**) The expression of the pluripotency marker proteins OCT4, SALL4, SOX2, KLF4, and ZFP206 in ESC BTL1 cells is demonstrated by immunoblotting. The expression of α-Tubulin is shown as loading control.

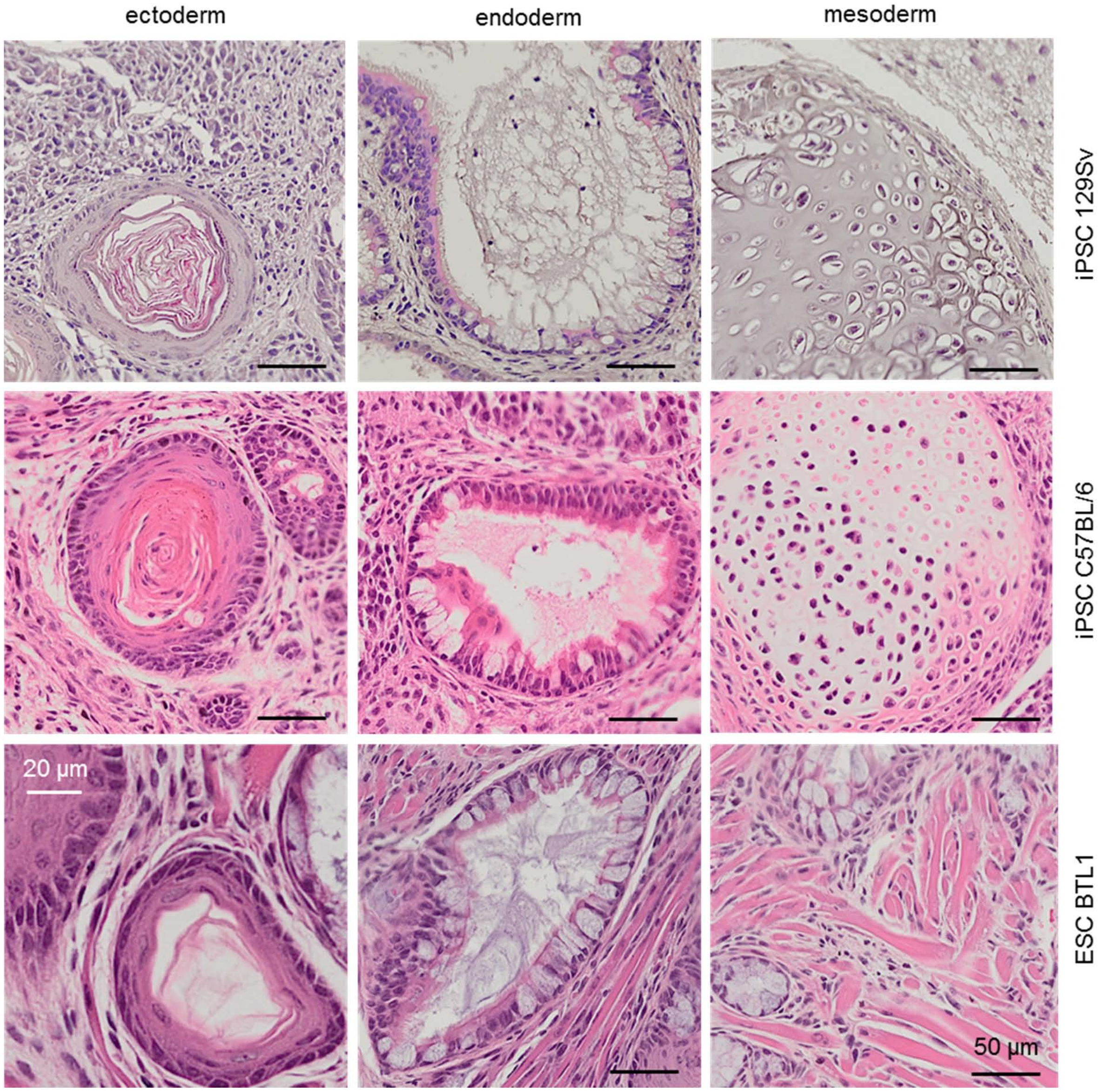

Supplementary Figure 4. Cells of the stem cell lines iPSC 129Sv, iPSC C57BL/6, and ESC BTL1 were subcutaneously injected into immunodeficient SCID/beige mice and resulting tumors were sectioned and stained with H&E. For each cell line, an ectodermal differentiation (keratinized epithelium), endodermal differentiation (intestinal epithelium), and mesodermal differentiation (cartilage or muscle cells) is shown. The black scale bars indicate 50 µm and the white scale bar 20 µm.

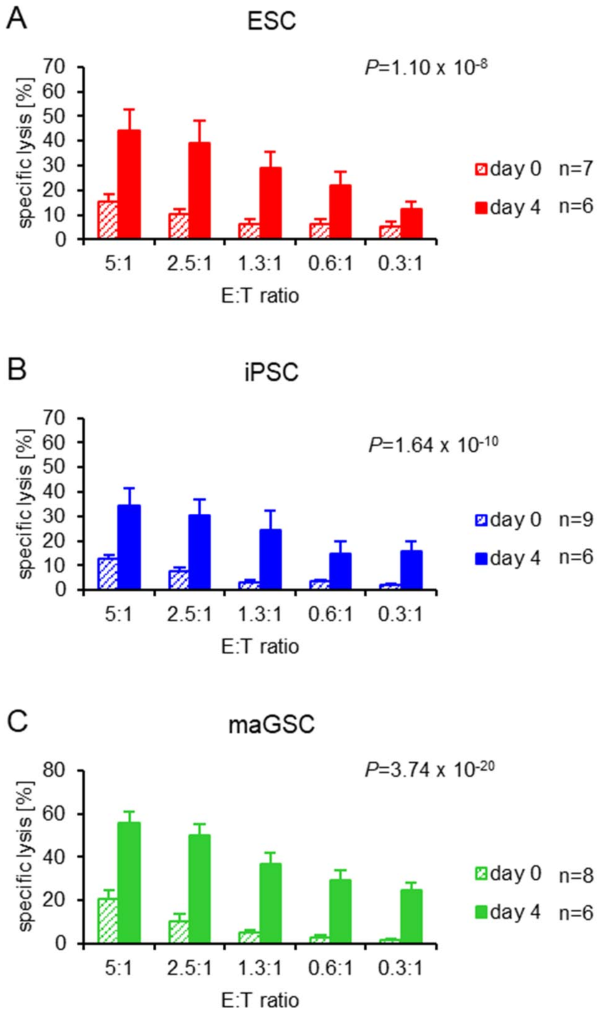

Supplementary Figure 5. A summary of means and SEM of specific lysis of (**A**) ESCs, (**B**) iPSCs, and (**C**) maGSCs by freshly purified NK cells (day 0) or IL-2-activated NK cells (day 4) from C57BL/6 wild type mice is shown as determined by ^{51}Cr-release assays. *P*-values for the comparisons (2-way-ANOVA adjusted for E:T ratios) are indicated for the comparison of killing by resting and IL-2-activated NK cells.

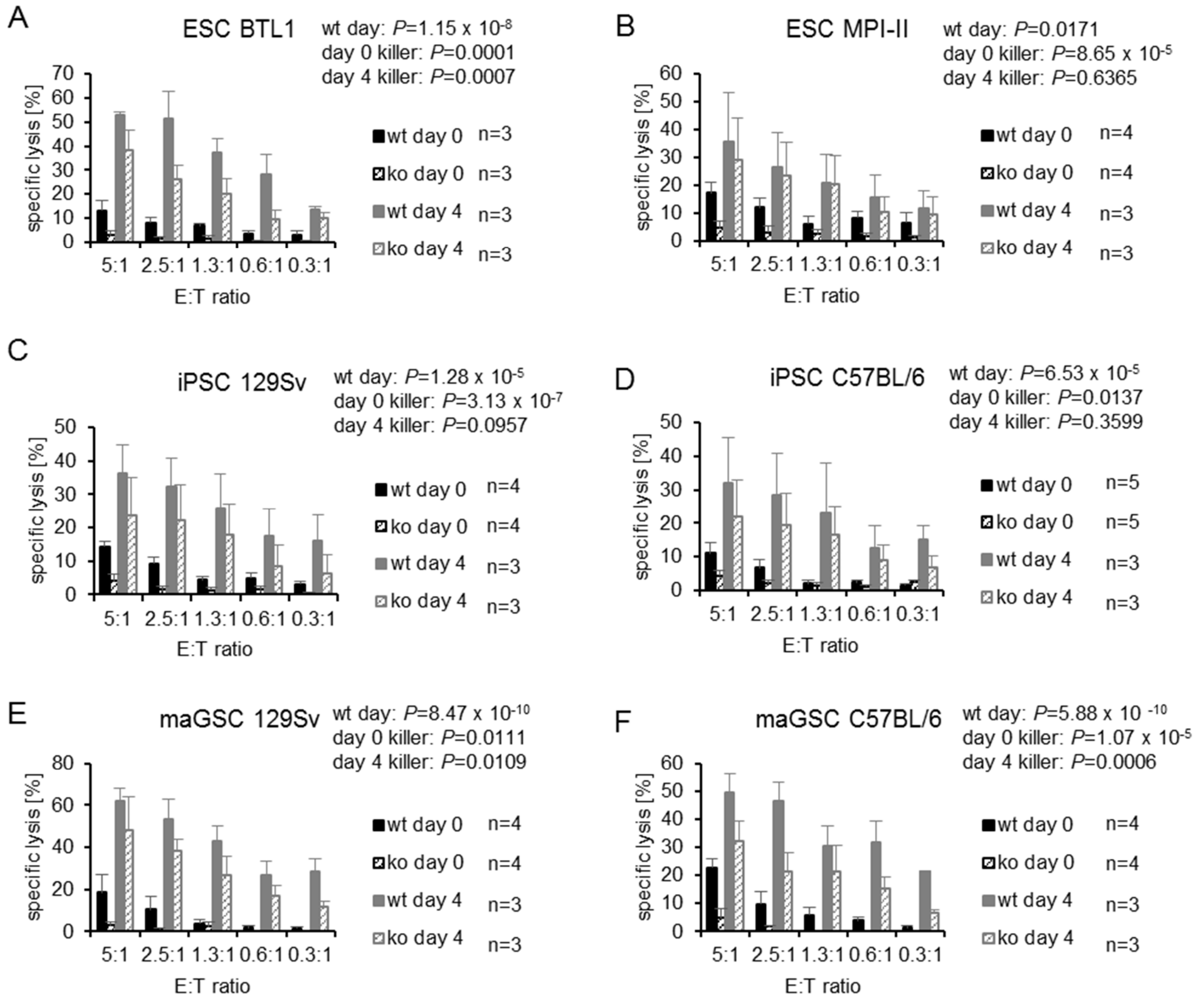

Supplementary Figure 6. A summary of means and SEM of specific lysis of (**A**) ESC BTL1 cells, (**B**) ESC MPI-II cells, (**C**) iPSC 129Sv cells, (**D**) iPSC C57BL/6 cells, (**E**) maGSC 129Sv cells, and (**F**) maGSC C57BL/6 cells by freshly purified NK cells (day 0) or IL-2-activated NK cells (day 4) from C57BL/6 wild type (wt) or *Klrk1*$^{-/-}$ mice (ko) is shown as determined by ^{51}Cr-release assays. *P*-values (2-way-ANOVA adjusted for E:T ratios) are indicated for the comparison of killing by resting and wild type NK cells (wt day) as well as by resting wild type and NKG2D-deficient NK cells (day 0 killer) and IL-2-activated wild type and NKG2D-deficient NK cells (day 4 killer).
